# Systemic Vascular Dysregulation May Be Associated With Lower Peripapillary Vessel Density in Non-glaucomatous Healthy Eyes: A Prospective Cross-Sectional Study

**DOI:** 10.3389/fmed.2021.678829

**Published:** 2021-08-27

**Authors:** Yiqin Guo, Yunxiao Sun, Haoran Liu, Kai Cao, Ningli Wang

**Affiliations:** ^1^Beijing Tongren Eye Center, Beijing Tongren Hospital, Capital Medical University, Beijing, China; ^2^Beijing Institute of Ophthalmology, Beijing Tongren Hospital, Capital Medical University, Beijing, China; ^3^Department of Medical Genetics, Capital Institute of Pediatrics, Beijing, China

**Keywords:** non-glaucomatous healthy eyes, peripapillary vessel density, nailfold capillaroscopy, cold provocation test, Flammer Syndrome, systemic vascular dysregulation

## Abstract

**Objective:** To determine whether systemic vascular dysregulation (SVD) evaluated by nailfold capillaroscope and Flammer Syndrome Questionnaire (FSQ) affects retinal peripapillary microcirculation in non-glaucomatous healthy eyes at steady status.

**Methods:** 120 healthy eyes from 63 non-glaucomatous subjects underwent Optical coherence tomography angiography (OCTA) after a rest of 30 minutes. Average retinal peripapillary capillary (RPC) vessel density (VD) and sectoral VD were automatically calculated, and peripapillary retinal nerve fiber layer thickness (RNFLT) was measured. Vasospastic diathesis was assessed using Flammer Syndrome Questionnaire (FSQ). Cold provocation test (CPT) was performed using nail-fold capillaroscope after OCTA. Positive CPT and a score of FSQ higher than 20% were necessary to determine a subject with SVD. Systemic and ocular parameters were compared between subjects with and without SVD.

**Results:** In this study, heart rate (*p* = 0.042), ocular perfusion pressure (*p* = 0.014) and average RPC vessel density (*p* = 0.046) was significantly different between subjects with and without SVD determined by the combination of CPT and FSQ. Generalized estimation equation (GEE) showed lower VD was significantly associated with longer axial length (β = −0.352, *p* = 0.001), thinner peripapillary retinal nerve fiber layer thickness (RNFLT) (β = 0.296, *p* < 0.001), SVD determined by CPT and FSQ (β = 0.617, *p* = 0.003) and high blood pressure (β = −0.879, *p* < 0.001). In the superotemporal sector, multivariate model showed only SVD was associated with RPC vessel density (β = −0.811, *p* < 0.001).

**Conclusion:** In subjects with SVD and non-glaucomatous healthy eyes, lower RPC vessel density in the superotemporal sector was observed. SVD determined by CPT and FSQ was significantly associated with attenuated retinal peripapillary microcirculation. Studies on ocular diseases relevant to vasospasms should consider the effects of SVD on the retinal peripapillary capillaries.

## Introduction

Systemic vascular dysregulation (SVD) is a condition of inappropriate constriction or insufficient dilation of the microvasculature in response to irritation like cold provocation or emotional stress (1). People who suffer from SVD often experience variant degrees of migraine, cold extremities, and low blood pressure (BP) (2, 3). SVD has been reported to be a risk factor for anterior ischemic optic neuropathy, retinal venous occlusion (4), central serous chorioretinaopathy (5), and especially for open angle glaucoma (6). Increased retinal venous pressure (7), glaucomatous optic disc with polar notching (8, 9), and retinal glial cell activation (10) were found to be associated with SVD. It was speculated that the vasospasm in the retinal microcirculation might produce higher local vascular resistance for the perfusion of the retinal nerve fiber layer inducing secondary loss of the RNFL and ganglion cells (11). However, in glaucoma patients, the effect of SVD on the peripapillary vasculature in the retina has been always confounded by the secondary impairment from the retinal nerve fiber layer loss. Thus, it is important to study the characteristics of the impact of SVD on the peripapillary microcirculation in non-glaucomatous healthy eyes. In addition, there were studies demonstrated that the peripapillary vessel density (VD) in the unaffected eyes (12) or unaffected hemifield (13) of the POAG patients was higher than the VD in their fellow affected eyes or the affected hemifield, but it was lower than the VD in the healthy eyes. These studies were (1, 2)conducted under normal condition of the subjects rather than under cold provocation. We speculate that these differences were caused by the higher prevalence of SVD in the POAG group than in the healthy group (14, 15) and that the extent of vasoconstriction in the peripapillary area under steady condition was higher in the subjects with SVD than the ones without. However, at present, there is a lack of study that can help testify possible influence associated with SVD in the peripapillary retinal capillaries under steady state in non-glaucomatous healthy eyes. To our knowledge, this is the first time to fill in this gap and provide sectoral differences of VD measured by OCTA.

Nailfold capillaroscopy (NFC), a non-invasive convenient technology, can provide visual inspection of dynamic activities of the peripheral capillaries with high magnification and resolution for the observation of blood cells in the living body. Digital microcirculation was associated with retinal microcirculation (16). Cold provocation test (CPT) performed by NFC was introduced to determine vasospasm as the most accurate method (17).

Optical coherence tomography angiography (OCTA) is a noninvasive effective device that can assess the retinal capillary network with high repeatability (18). Radial Peripapillary Capillary (RPC) vessel density (VD) evaluated by automatic metrics of OCTA can be a sensitive parameter for early detection of the predisposed glaucomatous changes (12, 19).

In this study, we used CPT combined with Flammer Syndrome Questionnaire (3, 20) to separate people with and without SVD and evaluate the association between SVD and retinal structural and vascular changes in non-glaucomatous healthy eyes.

## Methods

### Participants

All the participants were recruited from an ongoing prospective study (Chinese Clinical Trial Registry No. ChiCTR1800017875) approved by the Ethics Committee of Beijing Tongren Hospital (No. TRECKY2018-044) from November 2020 to February 2021. The subjects were volunteers from a neighbor community, staff of the hospital, or medical students. The study adhered the tenets of the declaration of Helsinki and all participants provided written informed consent after entering the research. The inclusion criteria were (1) subjects with non-glaucomatous healthy eyes confirmed by normal result of optical coherence tomography, Humphrey visual field test and non-contact tonometer; (2) no history of ocular trauma or intraocular surgery, any optic nerve or retinal pathology; (3) spherical diopter between +3.0 to −8 diopters (D). The exclusion criteria were (1) usage of anti-hypertensive drug and anticoagulant, (2) history of connective tissue diseases, diabetes mellitus, hypertension, hypercoagulation state, or other severe systemic diseases that could influence the retinal or digital circulation except for hyperlipidemia, (3) history of non-dominant hand trauma within 1 month, (4) poor clarity in site of nailfold, (5) optic disc tilt (ratio of the longest to the shortest diameter of the optic disc) higher than 1.3, (6) vitreous opacity or cataract and (7) glaucoma suspect.

Systemic parameters were obtained on the day of testing including age, sex, systolic blood pressure (SBP), diastolic blood pressure (DBP), heart rate (HR). Blood pressures were measured at the sitting position after 10 minutes of rest using an electronic hemopiezometer (Omron, HEM-7211, Japan). High blood pressure (HBP) was defined as blood pressure higher than 140/90 mmHg on the visiting day. The mean arterial pressure (MAP) were calculated as the equation: MAP = DBP+ 1/3(SBP- DBP). Ocular perfusion pressure (OPP) were calculated as the equation: OPP = 2/3MAP – IOP. Comprehensive ophthalmic examination was performed for every participant including: optometry test, best corrected visual acuity, intra ocular pressure (IOP), axial length and central corneal thickness (Lenstar LS 900, Haag-Streit Koeniz, Switzerland), fundus color photography (Nonmyd WX 3D, Kowa Company Ltd, Japan), OCTA (AngioVue; Optovue, Fremont, CA), OCT (RTVue-XR Avanti, Optovue, Fremont, CA) and visual field test (Humphery Field Analyzer II 750, Swedish interactive threshold algorithm, SITA fast 24-2, Carl Zeiss Meditec).

### Determination of Systemic Vascular Dysregulation

#### Flammer Syndrome Questionnaire

**Figure 1:**
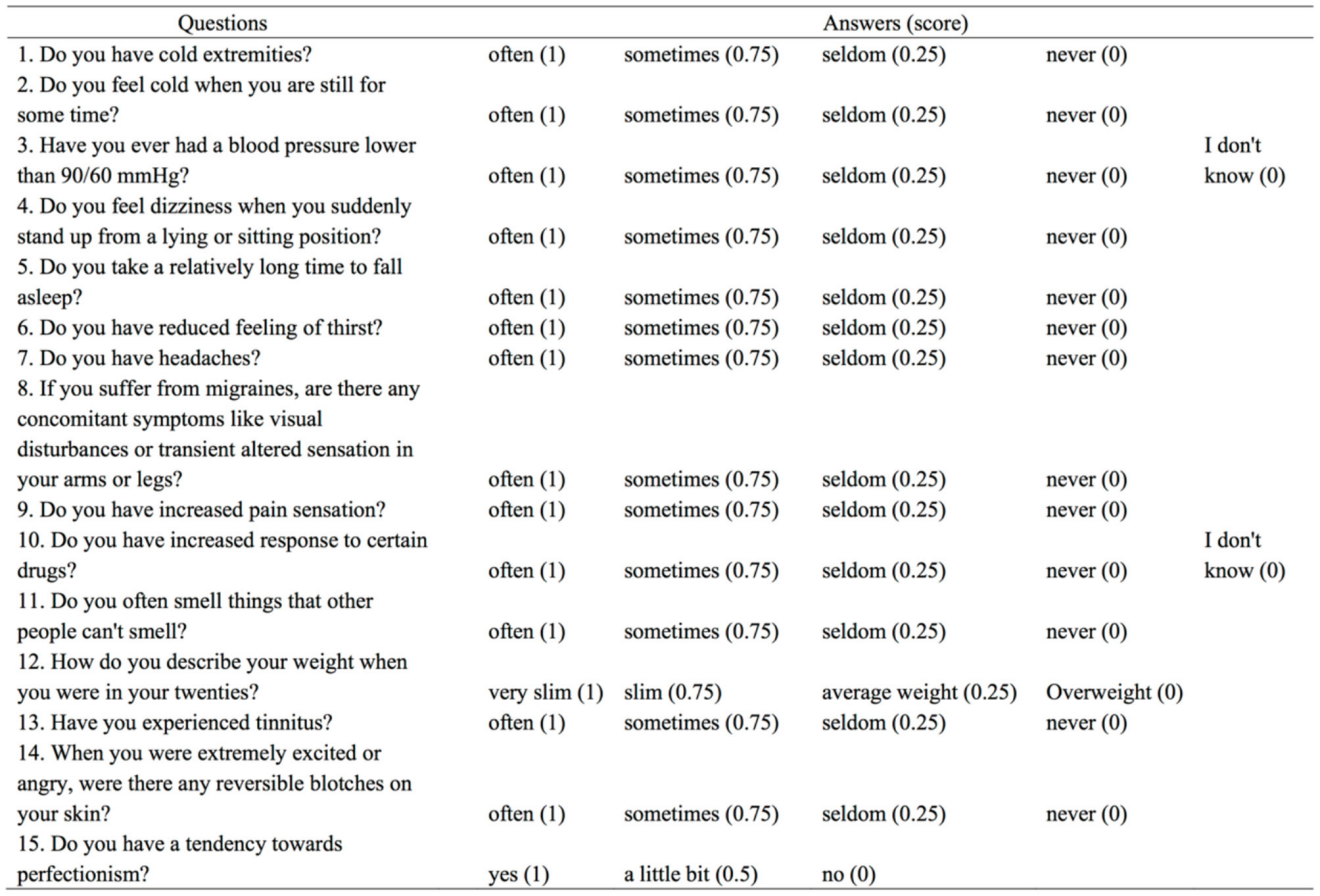
Questions and scoring of Flammer Syndrome Questionnaire.

Every participant was asked to answer the Flammer Syndrome Questionnaire (FSQ) (Figure 1) regarding the vasospastic symptoms. To evaluate the extent of subjective vasospastic experiences, the answers were categorized into “often”, “sometimes”, “seldom”, “never” and “I don't know”, which were graded into the score of 1, 0.75, 0.25, and 0. The scores of FSQ were calculated into the form of percentage after excluding the answers of “I don't know”.

#### Cold Provocation Test by Nailfold Capillaroscopy

The cold provocation test (CPT) was performed by nailfold capillaroscopy (INSPECTIS, S-028, Sweden) after the OCTA test to avoid temporary autonomic system irritation. Before the examination, the participants were asked to rest in the laboratory for at least 30 minutes at the room temperature of 25°C. To avoid the disturbance of morphology and obtain the most precise evaluation, the CPT test was performed on the fourth finger of the non-dominant hand after smearing with almond oil on the nailfold area. The videos of the nailfold capillaries were recorded at 300× magnification. After adjusting the hand to the same height of heart, the first video clip was recorded for 1 minute as contrast. Every part of the first row of capillaries along the nailfold was recorded for at least 5 seconds to observe baseline blood flow velocity. Later, the finger was immersed into 40°C warm water for 3 minutes and the second video clip was recorded for 1 minute. The recording method was the same as the first video clip. Last, the third video was recorded after immersion of −5°C ice saline for 1 minute. Each part of the first row of capillaries along the nailfold was quickly recorded to capture a blood flow standstill. The recording was ended until the blood flow recovered. Finally, the tested finger was gently covered by a soft cloth to recover to normal temperature after the end of the examination. Two observers (YQ.G and YX.S) identified whether there was a blood flow standstill (stops for more than 12 seconds) 2 weeks later after the videos were blinded to the clinical information.

SVD was identified as long as a patient's FSQ score higher than 20% and have positive CPT at the same time.

#### OCT Angiography Acquisition

The OCTA images were obtained by a commercially available device (AngioVue; Optovue). Every image contains 1 vertical and 1 horizontal priority of raster volumetric pattern with an 840 × 10 nm scan beam wavelength. Structural and blood flow information of high-definitional quality was acquired simultaneously at a 4.5 × 4.5 mm square centered on the optic disc. The optic disc margin was determined manually on the B-scan before measuring the parameters. The measurement of VD extends from the internal limiting membrane (ILM) to the posterior boundary of RNFL using system-provided AngioVue software (version 2017). Retinal vessel density was calculated as the percentage area of the vessel signals in a 750-μm-wide elliptical annulus stretching from the margin of the optic disc. The RNFLT was calculated in a band of 1 mm width extending from the optic disc boundary.

Poor quality images were excluded: (1) strength index less than 47; (2) motion artifacts; (3) wrong RNFL segmentation.

### Statistical Analysis

Inter-observer agreement on the identification of positive CPT was assessed using kappa statistics (κ = 0.866). Any disagreement was solved by the third person (Y.Z). Comparisons of systemic parameters between 2 groups were performed using independent *t*-tests, Mann-Whitney U-test, or Chi-square test. Generalized estimation equation (GEE) was used to compare ocular variables and test the factors associated with VD accounting for inter-eye correlations. In univariate regression analysis, variables included in the model were as follows: age, sex, FSQ scores, SBP, DBP, HR MAP, OPP, IOP, axial length, CCT, average RNFLT, HBP, HBC, and SVD determined by FSQ and CPT. The multivariate model included parameters with *P-*value <1.5 in univariate regression analysis. Correlation analyzes between RPC vessel density and RNFLT, axial length, and FSQ score were performed using Spearman analysis. All statistical analyses were performed by Statistical Package for the Social Sciences (version 22.0, Chicago, IL, USA). *P* values <0.05 were considered statistically significant. All data are presented as mean and standard deviation.

## Result

### Demographics and Clinical Characteristics

This study included 63 participants, among which 15 were identified as SVD (+) by positive CPT and an FSQ score higher than 20% (Table 1). The mean age of SVD (–) and SVD (+) were 43.33 ± 16.26 and 41.47 ± 13.33 years respectively. No significant difference was found in age, high blood pressure and history of hyperlipidemia between these two groups (Table 1). Unlike SVD (–), in SVD (+) there were more female than male subjects, but no significant difference in sex was found between the two groups. There was no significant difference in the mean systolic pressure (*p* = 0.056), diastolic pressure (*p* = 0.196), and mean arterial pressure (*p* = 0.056) between the SVD (+) and SVD (–) group. The SVD (+) has significantly lower heart rate (*p* = 0.042), higher FSQ scores (*p* < 0.001) and lower ocular perfusion pressure (*p* = 0.014) than SVD (–). There was no significant difference between the two groups in the spherical diopter, axial length, central cornea thickness, and intraocular pressure.

**Table 1:** Demographics and clinical characteristics.

	**SVD (–)**	**SVD (+)**	***P* value**
	**(*n* = 48)**	**(*n* = 15)**	
Age (years)	43.33 ± 16.26	41.47 ± 13.33	0.686^†^
Sex (male/female)	25/23	5/10	0.204^‡^
Systolic pressure (mmHg)	125.57 ± 16.18	115.83 ± 13.98	0.056^*^
Diastolic pressure (mmHg)	79.33 ± 11.58	74.97 ± 7.08	0.196^*^
Heart rate (times per minute)	77.46 ± 11.55	70.87 ± 7.48	0.042^*^
Mean arterial pressure (mmHg)	94.75 ± 12.45	85.48 ± 15.15	0.056^†^
Ocular perfusion pressure (mmHg)	48.07 ± 8.41	41.11 ± 9.8	**0.014** ^†^
Flammer syndrome questionnaire (%)	19.56 ± 13.68	31.89 ± 9.37	**<0.001** ^†^
Spherical diopter (diopters)	−1.81 ± 2.24	−2.48 ± 2.15	0.262^†^
Axial length (mm)	24.28 ± 1.33	24.42 ± 1.08	0.646^†^
Central cornea thickness (mm)	534.45 ± 33.61	529.24 ± 25.00	0.616^*^
Intraocular pressure (mmHg)	15.26 ± 2.56	15.71 ± 1.89	0.376^*^
Retinal nerve fiber layer thickness (μm)	111.81 ± 10.39	113.43 ± 11.96	0.562^*^
Average RPC vessel density	52.02 ± 2.56	50.32 ± 3.46	**0.046** ^†^
High blood pressure (n, %)	7, 14.6	1, 6.7	0.719^‡^
Hyperlipidemia (n, %)	8, 16.7	0, 0	0.212^‡^

*RPC, radial peripapillary capillary; SVD, systemic vascular dysregulation*.

* *The P value was determined by independent t-test*.

† *The P value was determined by Mann-Whitney U-test*.

‡ *The P value was determined by Chi-square test. Boldface P values indicate statistically significant difference at p < 0.05*.

### Comparison of Peripapillary Retinal Nerve Fiber Layer Thickness and Peripapillary Retinal Vessel Density in Different Sectors

The mean peripapillary RNFLT of the SVD (–) and SVD (+) were 111.81 ± 10.39 and 113.43 ± 11.96 respectively (p = 0.562) (Table 2). The average peripapillary retinal vessel density was 52.02 ± 20.56 and 50.32 ± 30.46 (*p* = 0.046). There was no significant difference in the sectoral comparison of the peripapillary RNFLT (Table 2). However, the SVD (+) has lower peripapillary retinal vessel density in the superotemporal sector than SVD (–) group (*p* < 0.001).

**Table 2:** Comparison of peripapillary retinal nerve fiber layer thickness and RPC vessel density between subjects with and without SVD.

		**SVD (–)**	**SVD (+)**	***P* value**
		**(*n* = 91 eyes)**	**(*n* = 29 eyes)**	
RNFLT	TS	81.32 ± 13.04	86.62 ± 15.96	0.165^†^
	ST	142.10 ± 20.55	146.69 ± 23.17	0.399^†^
	SN	141.16 ± 22.10	134.29 ± 27.20	0.351^*^
	NS	95.45 ± 17.78	95.11 ± 24.50	0.872^*^
	NI	76.39 ± 14.83	81.76 ± 20.89	0.31^*^
	IN	138.99 ± 24.20	135.51 ± 23.01	0.547^*^
	IT	158.51 ± 19.41	167.06 ± 24.80	0.151^*^
	TI	78.22 ± 14.75	80.60 ± 16.59	0.483^†^
VD	TS	57.27 ± 3.11	55.59 ± 4.47	0.156^†^
	ST	55.49 ± 3.98	52.34 ± 3.46	**<0.001** ^†^
	SN	50.18 ± 3.89	48.75 ± 3.76	0.101^†^
	NS	47.69 ± 4.15	45.74 ± 5.18	0.117^†^
	NI	46.24 ± 4.83	45.24 ± 6.50	0.516^*^
	IN	51.08 ± 4.47	49.11 ± 4.49	0.104^*^
	IT	57.28 ± 4.44	55.87 ± 4.29	0.128^*^
	TI	54.67 ± 3.46	53.65 ± 5.87	0.422^†^

*RPC, radial peripapillary capillary; SVD, systemic vascular dysregulation; RNFLT, retinal nerve fiber layer thickness; VD, vessel density; TS, temporalsuperior; ST, superotemporal; SN, superonasal; NS, nasalsuperior; NI, nasalinferior; IN, Inferiornasal; IT, Inferiortemporal; TI, temporalinferior*.

* *The P value was determined by independent t-test*.

† *The P value was determined by Mann-Whitney U-test. Boldface P values indicate statistically significant difference after Bonferroni correction*.

### Generalized Estimation Equation Analyses for Factors Associated With Average Peripapillary Retinal Vessel Density

Using generalized estimation equation analysis, axial length (*p* < 0.001), average RNFLT (*p* < 0.001) and SVD determined by CPT and FSQ scores (*p* = 0.046) were significantly associated with average peripapillary retinal vessel density in univariate model (Table 3). Parameters with *P* value smaller than 0.15 were included in the multivariate model. Axial length (β = −0.352, *p* = 0.001), average RNFLT (β = 0.296, *p* < 0.001), high blood pressure (β = −0.879, *p* < 0.001) and SVD (β = −0.617, *p* = 0.003) were significant factors associated with average peripapillary retinal vessel density in the final model.

**Table 3:** Univariate and multivariate generalized estimation equation analyses for factors associated with average RPC vessel density.

	**Univariate model**			**Multivariate model**		
**Variables**	**β**	**95% Confidence interval**	***P*-value**	**β**	**95% Confidence interval**	***P*-value**
sex (1 = male, 2 = female)	−0.161	−1.47, 1.148	0.81			
Age	0.035	−0.014, 0.083	0.164			
Intraocular pressure	−0.152	−0.344, 0.041	0.123	0.016	−0.163, 0.195	0.863
Axial length	−0.833	−1.272, −0.394	**<0.001**	−0.352	−0.559, −0.145	**0.001**
Central cornea thickness	−0.002	−0.021, 0.017	0.833			
Average RNFLT	0.118	0.074, 0.161	**<0.001**	0.296	0.146, 0.445	**<0.001**
Systolic blood pressure	−0.025	−0.065, 0.014	0.209			
Diastolic blood pressure	−0.031	−0.09, 0.028	0.297			
Heart rate	−0.017	−0.067, 0.033	0.516			
Ocular perfusion pressure	−0.028	−0.09, 0.033	0.367			
High blood pressure	−2.063	−4.487, 0.361	0.095	−0.879	−1.301, −0.457	**<0.001**
Hyperlipidemia	1.346	−0.177, 2.87	0.083	−0.062	−0.65, 0.526	0.836
FSQ score	−0.019	−0.065, 0.028	0.429			
SVD by FSQ and CPT	−1.702	−3.372, −0.032	**0.046**	−0.617	−1.03, −0.204	**0.003**

*RNFLT, retinal nerve fiber layer thickness; FSQ, Flammer Syndrome questionnaire; SVD, systemic vascular dysregulation; CPT, cold provocation test. Parameters with P value <0.15 in the univariate model were included in the multivariate model. Boldface P values indicate statistically significant difference at p < 0.05*.

### Generalized Estimation Equation Analyses for Factors Associated With Peripapillary Retinal Vessel Density in the Superotemporal Sector

In the univariate model, axial length (*p* = 0.036), and SVD determined by CPT and FSQ scores (*p* < 0.001) were significantly associated with peripapillary retinal vessel density in the superotemporal sector (Table 4). Parameters with *P* value smaller than 0.15 were included in the multivariate model. Only SVD (β = −0.811, *p* < 0.001) was a significant factor associated with peripapillary retinal vessel density in the superotemporal sector in the final model.

**Table 4:** Univariate and multivariate generalized estimation equation analyses for factors associated with RPC vessel density in the superotemporal sector.

	**Univariate model**			**Multivariate model**		
**Variables**	**β**	**95% Confidence interval**	***P*-value**	**β**	**95% Confidence interval**	***P*-value**
sex (1 = male, 2 = female)	1.043	−0.662, 2.749	0.231			
Age	0.059	−0.006, 0.124	0.075	0.178	−0.101, 0.456	0.211
Intraocular pressure	−0.219	−0.499, 0.061	0.125	−0.031	−0.189, 0.127	0.699
Axial length	−0.612	−1.184, −0.039	**0.036**	−0.098	−0.318, 0.123	0.386
Central cornea thickness	0.001	−0.025, 0.027	0.927			
Average RNFLT	0.019	−0.021, 0.059	0.355			
Systolic blood pressure	−0.015	−0.067, 0.037	0.575			
Diastolic blood pressure	−0.063	−0.131, 0.006	0.073	−0.182	−0.371, 0.007	0.059
Heart rate	−0.04	−0.112, 0.031	0.271			
Ocular perfusion pressure	−0.024	−0.111, 0.064	0.597			
High blood pressure	−1.852	−5.357, 1.654	0.301			
Hyperlipidemia	2.19	−0.544, 4.924	0.116	0.032	−0.716, 0.779	0.934
FSQ score	−0.054	−0.13, 0.023	0.169			
SVD by FSQ and CPT	−3.2	−4.951, −1.45	**<0.001**	−0.811	−1.243, −0.378	**<0.001**

*RNFLT, retinal nerve fiber layer thickness; FSQ, Flammer Syndrome questionnaire; SVD, systemic vascular dysregulation; CPT, cold provocation test. Parameters with P value <0.15 in the univariate model were included in the multivariate model. Boldface P values indicate statistically significant difference at p < 0.05*.

### Generalized Estimation Equation Analyses for Factors Associated With Peripapillary Retinal Vessel Density in the Inferotemporal Sector

In the univariate model, axial length (*p* = 0.02), and hyperlipidemia (*p* = 0.016) were significantly associated with peripapillary retinal vessel density in the inferotemporal sector (Table 5). Parameters with *P*-value smaller than 0.15 were included in the multivariate model. No significant factor was found associated with peripapillary retinal vessel density in the inferotemporal sector in the final model.

**Table 5:** Univariate and multivariate generalized estimation equation analyses for factors associated with RPC vessel density in the inferotemporal sector.

	**Univariate model**			**Multivariate model**		
**Variables**	**β**	**95% Confidence interval**	***P*-value**	**β**	**95% Confidence interval**	***P*-value**
sex (1 = male, 2 = female)	−0.288	−2.139, 1.563	0.76			
Age	0.031	−0.035, 0.098	0.356			
Intraocular pressure	−0.266	−0.603, 0.071	0.122	0		
Axial length	−0.767	−1.413, −0.121	**0.02**	−0.127	−0.308, 0.055	0.172
Central cornea thickness	0.005	−0.028, 0.038	0.757			
Average RNFLT	0.016	−0.023, 0.055	0.417			
Systolic blood pressure	−0.039	−0.095, 0.017	0.171			
Diastolic blood pressure	−0.052	−0.166, 0.012	0.195			
Heart rate	−0.077	−0.166, 0.012	0.089	−0.165	−0.483, 0.153	0.308
Ocular perfusion pressure	−0.041	−0.119, 0.038	0.313			
High blood pressure	−1.708	−5.378, −1.963	0.362			
Hyperlipidemia	2.46	0.462, 4.45	**0.016**	0.351	−0.183, 0.885	0.198
FSQ score	0.011	−0.06, 0.083	0.76			
SVD by FSQ and CPT	−1.458	−3.337, 0.421	0.128	−0.282	−0.838, 0.274	0.32

*RNFLT, retinal nerve fiber layer thickness; FSQ, Flammer Syndrome questionnaire; SVD, systemic vascular dysregulation; CPT, cold provocation test. Parameters with P value <0.15 in the univariate model were included in the multivariate model. Boldface P values indicate statistically significant difference at p < 0.05*.

### Correlation Analysis Between RPC Vessel Density and RNFLT, Axial Length, FSQ Score

**Figure 2:**
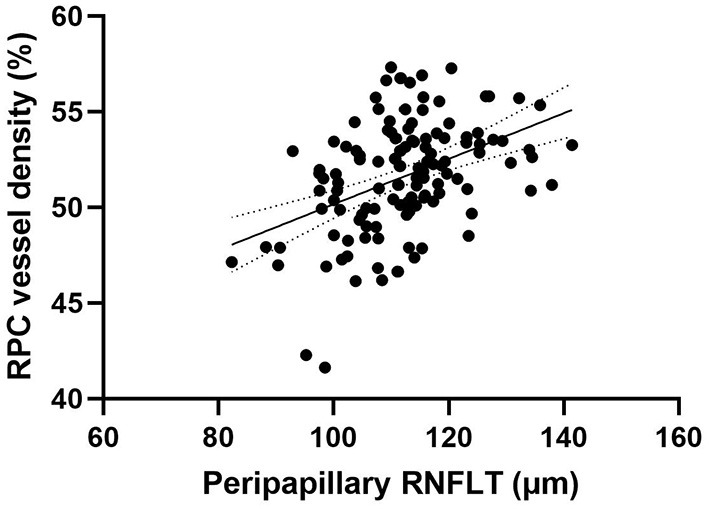
Correlation matrix between peripapillary retinal nerve fiber layer thickness and RPC vessel density performed by spearman analysis (*r* = 0.417, *p* < 0.001).

**Figure 3:**
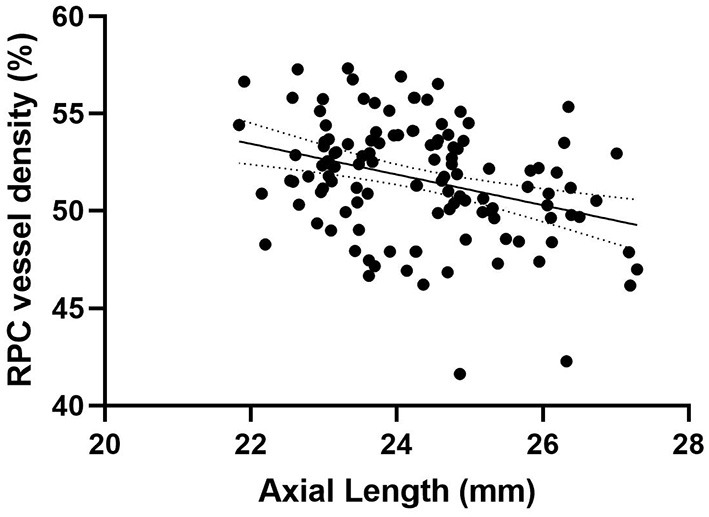
Correlation matrix performed by spearman analysis between Axial length and RPC vessel density (*r* = −0.317, *p* < 0.001).

**Figure 4:**
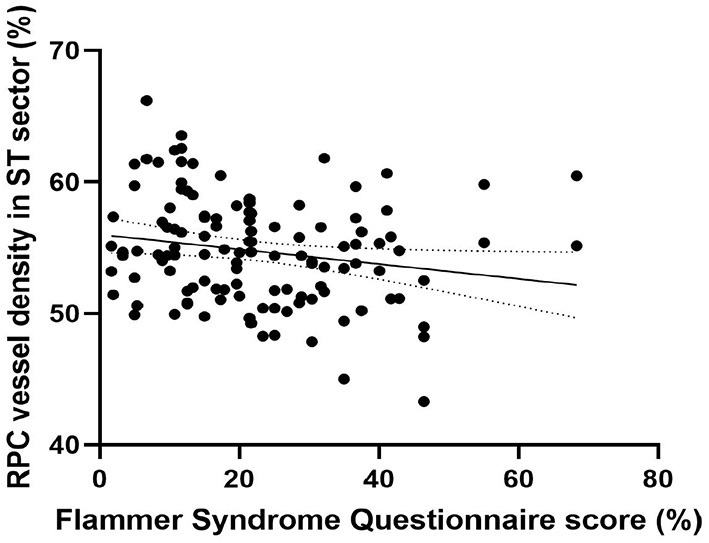
Correlation matrix performed by spearman analysis between Flammer Syndrome questionnaire score and RPC vessel density in the superotemperal sector (*r* = −0.214, *p* = 0.019).

Peripapillary retinal nerve fiber layer thickness (*r* = 0.417, *p* < 0.001) (Figure 2) and axial length (*r* = −0.317, *p* < 0.001) (Figure 3) was significantly associated with the average RPC vessel density, while FSQ score (*r* = −0.214, *p* = 0.019) (Figure 4) was significantly associated with RPC vessel density in the superotemperal sector.

## Discussion

Since the OCTA test was performed after a rest for 30 minutes and before the cold provocation test, the lower RPC vessel density in this study can be interpreted as attenuate retinal capillaries associated with vasospastic predisposition rather than irritation-induced vasoconstriction of the retinal arterioles. There was a study that demonstrated a significant decrease in the retinal capillary flow of patients with Raynaud's phenomenon during the immersion of a hand in the ice water, which remained 17% reduced for at least 10 minutes after removal (21). The question is, however, whether this reversible reaction can cause lasting impairment to the terminal capillaries of the retina or permanent injury to the retinal nerve fiber layers. The answer to this question is crucial to the relationship between vascular dysregulation and glaucoma. To our knowledge, this is the first study to compare the RPC vessel density on steady status in non-glaucomatous healthy eyes with and without vasospastic predisposition.

To be clear, several ocular factors have strong association with peripapillary vessel density in healthy eyes. Consistent with other studies (22–24), the longer the axial length, the lower the retinal VD in the peripapillary area. Reduced vessel density was observed with more myopic eyes. In this study, there was no significant difference in the axial length and spherical diopter between subjects with and without SVD. In addition, the thinner the retinal nerve fiber layer thickness, the lower the retinal VD distributed in the peripapillary area (22, 25, 26). This correlation demonstrates the normal balance of supply and demand in the optic nerve head. In this study, there was no significant difference neither in the average RNFLT nor in the sectoral analysis of RNFLT between subjects with and without SVD. Thus, the demand of blood supply to the retinal nerve fiber layer could be assumed similar. However, it is noticeable that the VD in the superotemporal sector was significantly lower in the SVD (+) than in SVD (–). This result may imply potential insufficient blood supply in this area in the SVD (+) group.

**Figure 5:**
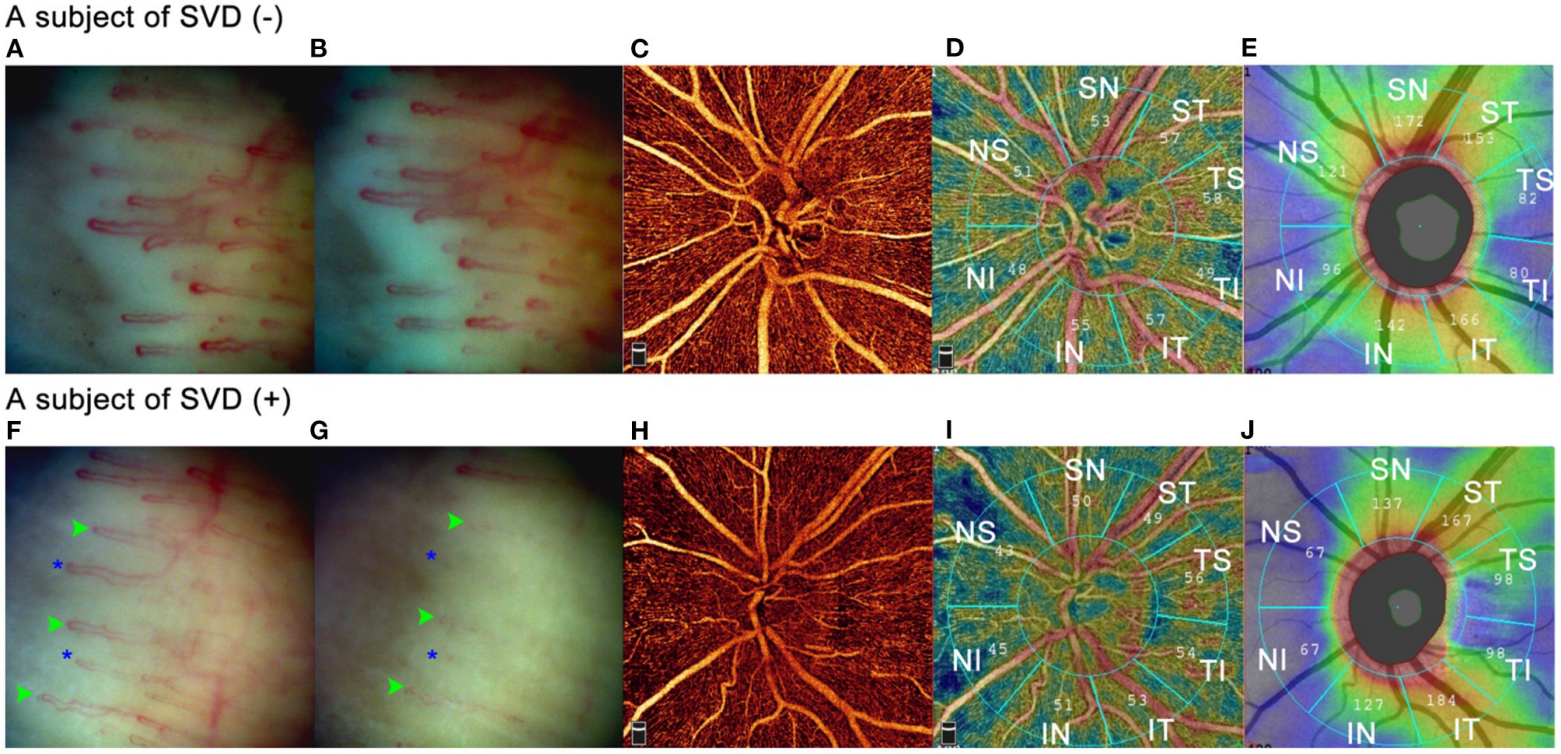
Comparisons of nailfold capillary cold provocation test and OCTA between subjects with and without SVD. Two subjects were illustrated in Figure 5. Image **(A–E)** were from a 34-year-old female without SVD. SBP/DBP was 118mmHg /81mmHg with a heart rate of 83 per minute. IOP was 15.9 mmHg. Image **(F–J)** were from a 42-year-old female with SVD. SBP/DBP was 127.5 mmHg/75 mmHg with a heart rate of 67 per minute. IOP was 15.2 mmHg. **(A)** and **(F)** showed nailfold capillaries after immersion in warm water for 3 minutes. **(B)** and **(G)** illustrated nailfold capillaries after immersion of ice saline at −5°C for 1 minute. No blood flow standstill was observed in the SVD (–) subject, whereas in the SVD (+) subject several capillaries showed blood cell standstill for 40 seconds (green arrowheads) and some capillaries disappeared (blue asterisks). **(C)** and **(H)** were RPC maps of OCTA. The SVD (+) subject showed sparser RPCs than the SVD (–) subject. **(D)** and **(I)** showed sectoral vessel density calculated by AngioVue software according to Garway-Heath partition. **(E)** and **(J)** illustrated sectoral RNFLT according to Garway-Heath partition. In the IN, NI, NS, and SN sectors, the SVD (+) subject had thinner RNFLT than the SVD (–) subject did, and the vessel density was lower in the SVD (+) than in the SVD (–) subject in these sectors. Whereas in the ST, TS, and IT sectors, although the SVD (+) subject had thicker RNFLT than the SVD (–) subject did, the vessel density was lower in the SVD (+) than in the SVD (–) subject in these sectors.

To further testify plausible factors affecting the average VD and sectoral VD sensitive to the diagnosis of glaucoma, we used Generalized estimation equation to perform univariate and multivariate regression analyzes and eliminate the inter-eye correlation. Besides axial length and RNFLT, average VD was negatively associated with HBP (*p* < 0.001) and SVD (*p* = 0.003) in the multivariate model. HBP patients in our study were subjects who presented blood pressure higher than 140/90 mmHg on the visiting day but without a medical history of hypertension. Temporary rise of blood pressure can cause retinal blood vessel changes by vasoconstrictive and malignant phases to control the volume of blood received by retinal capillary bed retinal arteriole (27–29). Contrary to HBP, subjects with SVD often have lower systolic and diastolic blood pressure and lower heart rate (2, 30). The lower average VD in the SVD may be resulted from a higher level of endothelin-1(ET-1) in the circulating blood (31) or enhanced response to vasoconstrictors (32). As can be seen in Figure 5, two examples showed comparisons of the nailfold capillary cold provocation test and OCTA images between subjects with and without SVD. The overall status of the retinal arterioles in subjects with vascular dysregulation tends to be more constrictive than people without. Although in one study (33) the retinal vascular diameter was not found significantly different in subjects with and without vasospastic propensity, the limitation was mentioned that only one pair of vessels within a defined distance from the optic disc was measured, thus the difference of vessels with the smaller caliber or those in the upper retina was omitted. Further studies are needed to testify the status of vasoconstriction of retinal arterioles in SVD at steady state. In our study, the lower vessel density may suggest decreased blood flow volume in the radial peripapillary capillaries.

Different from the average VD, the sectoral VD in the superotemperal peripapillary area was only associated with SVD in the multivariate model. In addition, in this sector, there was no significant difference in the RNFLT between SVD (+) and SVD (–). This may imply that there was heterogeneous topographical peripapillary retinal blood supply in the SVD (+) group. Interestingly, the peripapillary superotemperal sector is one of the impressionable areas of glaucomatous damage. Several hints implied that impairment of retinal circulation might precede the loss of retinal nerve fiber layer loss. Chen CL (13) compared the normal hemisphere of eyes with glaucoma and normal eyes and discovered significant difference in vessel area density (*p* = 0.003) and blood flux index (*p* < 0.001), whereas RNFLT (*p* = 0.48) was not significantly different. Hou H (19) compared glaucoma suspect and healthy subjects and found a significant difference in the inter-eye asymmetry of vessel density in optic nerve head (*p* = 0.014) but not in the inter-eye asymmetry of structural thickness (*p* = 0.943). The higher prevalence of migraine and cold extremities in open angle glaucoma, especially normal tension glaucoma, is also evidence of possible effect of vascular dysregulation on the retina (14, 20). However, longitudinal studies are needed to testify if vasospasm-related peripapillary attenuated vasculature can result in a loss of RNFL in the future. In the clinical practice, a tendency toward SVD and the disproportionate or topographically non-corresponding decrease of peripapillary VD compared to the RNFLT loss may suggest vasospasm-related reduced retinal circulation. No factors were detected to be associated with sectoral VD in the inferotemporal peripapillary area in the multivariate model. More investigations with larger sample sizes and more parameters are needed for further research.

Cold provocation test by nailfold capillaroscopy as one of the objective examinations with binary outcome may occupy an important position in diagnosing vascular dysregulation. Different from complaints of patients regarding migraine, it is influenced little by confusion of depression or other types of cephalalgia, which might need further neurological examination for differential diagnosis. Its binary outcome can provide discriminable results, unlike other devices like dynamic vessel analyzes without a decision point. The difference of blood flow standstill time among healthy, normal tension glaucoma and high-tension glaucoma has been demonstrated before. The longer the standstill time of the blood flow, the more deteriorative the mean visual sensitivity was (15). Our study suggested possible local insufficiency in the retinal circulation on steady status in subjects diagnosed with SVD by both positive CPT and high FSQ scores. This result may reveal a lasting effect of systemic vascular dysregulation on retinal microcirculation.

There were limitations in this study. First, bias resulted from a small sample size should be considered. Due to the lower prevalence of SVD in healthy population than in glaucoma, a bigger sample size is needed to collect data from healthy subjects with SVD. Second, other systemic factors including analysis of blood compositions and hemodynamic parameters were not included in this study. Further investigations are needed to include the influence of these factors. Third, this is a cross-sectional study. Prospective longitudinal studies are needed to clarify the causal relationship between SVD and peripapillary retinal circulation and possible structural changes.

In conclusion, we found lower superficial peripapillary retinal vessel densities in subjects with systemic vascular dysregulation and non-glaucomatous healthy eyes, especially in the superotemporal sector. This is the first study to demonstrate that systemic vascular dysregulation may be associated with attenuated peripapillary retinal microcirculation on steady status. Thus, our study will be useful for further investigations of vascular impairment in ocular diseases associated with SVD. In addition, systemic vascular dysregulation profiling may be useful for early detection of the primary vascular impairment of the peripapillary vasculature.

## Data Availability Statement

The raw data supporting the conclusions of this article will be made available by the authors, without undue reservation.

## Ethics Statement

The studies involving human participants were reviewed and approved by the Ethics Committee of Beijing Tongren Hospital. The patients/participants provided their written informed consent to participate in this study.

## Author Contributions

YG and NW: study design. HL and KC: data analysis. YG and YS: writing and data collection. All authors contributed to the article and approved the submitted version.

## Conflict of Interest

The authors declare that the research was conducted in the absence of any commercial or financial relationships that could be construed as a potential conflict of interest.

## Publisher's Note

All claims expressed in this article are solely those of the authors and do not necessarily represent those of their affiliated organizations, or those of the publisher, the editors and the reviewers. Any product that may be evaluated in this article, or claim that may be made by its manufacturer, is not guaranteed or endorsed by the publisher.
