# Supplementary figures and images for: Systemic Vascular Dysregulation May Be Associated With Lower Peripapillary Vessel Density in Non-glaucomatous Healthy Eyes: A Prospective Cross-Sectional Study
Source: Front Med (Lausanne). 2021 Aug 27;8:678829. doi: 10.3389/fmed.2021.678829 (PMC8432616; doi:10.3389/fmed.2021.678829)

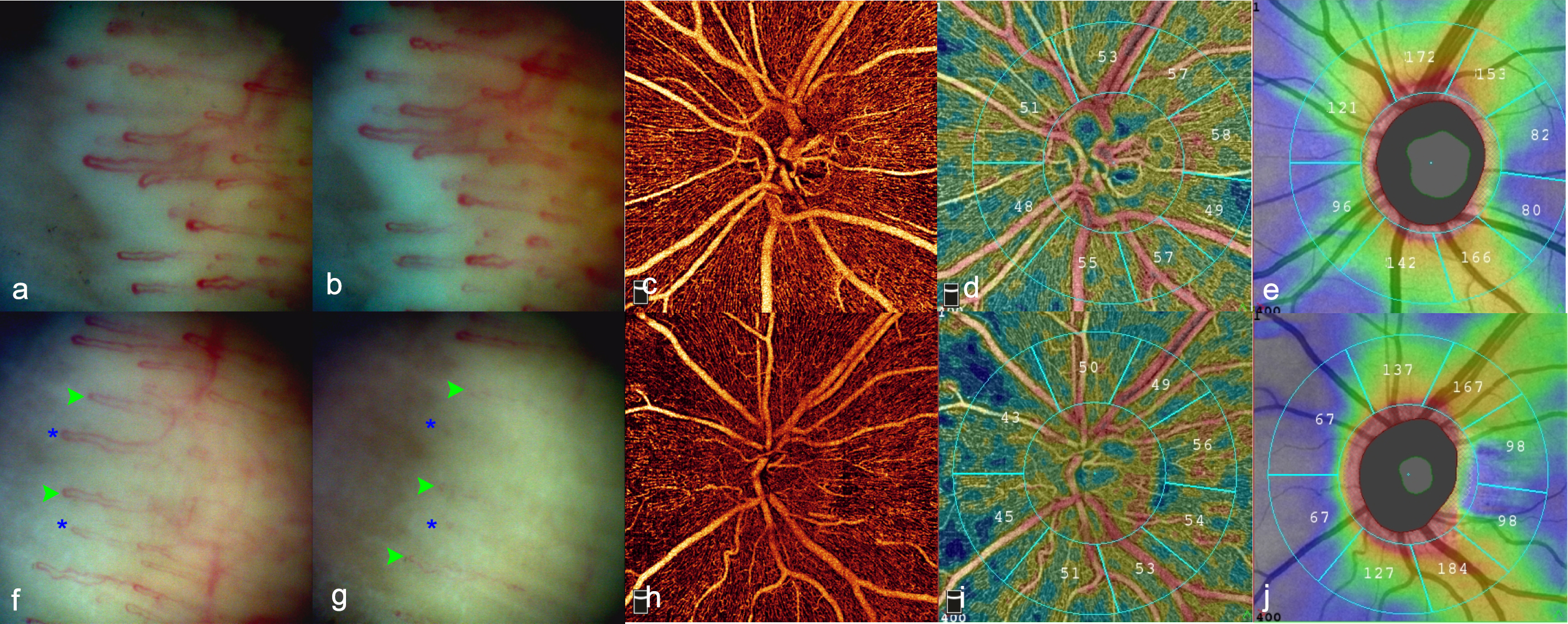

Supplement: Supplementary file 1 [file Image_1.JPEG]
